# Smoking Behaviors in Survivors of Smoking-Related and Non–Smoking-Related Cancers

**DOI:** 10.1001/jamanetworkopen.2020.9072

**Published:** 2020-07-02

**Authors:** Ellen R. Gritz, Rajesh Talluri, Joël Fokom Domgue, Irene Tami-Maury, Sanjay Shete

**Affiliations:** 1 Department of Behavioral Science, The University of Texas MD Anderson Cancer Center, Houston; 2 Department of Data Science, The University of Mississippi Medical Center, Jackson; 3 Department of Epidemiology, The University of Texas MD Anderson Cancer Center, Houston; 4 Department of Gynecologic Oncology and Reproductive Medicine, The University of Texas MD Anderson Cancer Center, Houston; 5 Department of Epidemiology, Human Genetics, and Environmental Sciences, The University of Texas Health Science Center at Houston School of Public Health, Houston; 6 Department of Biostatistics, The University of Texas MD Anderson Cancer Center, Houston; 7 Division of Cancer Prevention and Population Science, The University of Texas MD Anderson Cancer Center, Houston

## Abstract

**Importance:**

The population of cancer survivors is rapidly growing in the US. Tobacco smoking is associated with many cancers; however, whether cigarette smoking behaviors among cancer survivors vary according to cancer type—that is, smoking-related cancers (SRCs) vs non–smoking-related cancers (NSRCs)—remains unclear.

**Objectives:**

To examine cigarette smoking prevalence and behaviors (ie, continuing or quitting smoking) among cancer survivors and to compare them between survivors of SRCs and NSRCs.

**Design, Setting, and Participants:**

This study was a cross-sectional analysis of the 2017 National Health Interview Survey, a household survey of civilian US residents who were aged 18 years or older. The National Health Interview Survey is population based and is representative of the US population. Data analysis was performed from June to October 2019.

**Main Outcomes and Measures:**

The primary outcomes were prevalence of current cigarette smoking among cancer survivors and prevalence of continuing smoking and quitting smoking after a cancer diagnosis. Secondary outcomes included factors associated with continued smoking vs quitting smoking after a cancer diagnosis.

**Results:**

A total of 26 742 respondents (mean [SD] age, 50.97 [18.61] years; 14 646 women [51.76%]) to the 2017 National Health Interview Survey were included in this study. Of the 3068 individuals (9.42%) in the study population who had cancer, 589 (19.96%) were SRC survivors, 2297 (74.50%) were NSRC survivors, 168 (4.96%) were survivors of both SRC and NSRC, and the remaining 14 (0.58%) had missing information about the type of cancer. Four hundred forty-nine SRC survivors (54.08%) were women, compared with 1412 NSRC survivors (54.30%). Ninety-six SRC survivors (15.69%) and 151 NSRC survivors (7.99%) were younger than 45 years. Overall, 372 cancer survivors (13.16%) were current smokers. Current smoking prevalence was higher among survivors of SRCs (145 survivors [19.78%]) compared with NSRC survivors (251 survivors [10.63%]). Among cancer survivors, 309 current smokers at cancer diagnosis (43.96%) reported having successfully quit smoking and 372 (56.04%) reported continuing smoking. Among the continuing smokers, 176 (56.49%) reported an unsuccessful quit attempt in the last 12 months. After cancer diagnosis, SRC survivors had higher odds of continued smoking compared with NSRC survivors (odds ratio [OR], 2.10; 95% CI, 1.12-3.93; *P* = .02). Men (OR, 1.93; 95% CI, 1.05-3.57; *P* = .04), those with angina pectoris (OR, 5.40; 95% CI, 1.33-21.91; *P* = .02), and those with chronic bronchitis (OR, 2.55; 95% CI, 1.05-6.19; *P* = .04) had higher odds of continued smoking, whereas Hispanic participants (compared with non-Hispanic white participants: OR, 0.18; 95% CI, 0.05-0.68; *P* = .01) and married participants (compared with never married participants: OR, 0.33; 95% CI, 0.12-0.96; *P* = .04) had lower odds of continued smoking.

**Conclusions and Relevance:**

These findings suggest that compared with NSRC survivors, SRC survivors may be at higher risk of being cigarette smokers at cancer diagnosis and of continuing smoking afterward. Although smoking cessation interventions are critically important for all cancer survivors, special efforts should target survivors of SRCs.

## Key Points

**Question:**

Among adult cancer survivors, do cigarette smoking behaviors vary according to whether the cancer was smoking related or not smoking related?

**Findings:**

This cross-sectional study found that, in the 2017 US National Health Interview Survey, which included 26 742 respondents aged 18 years or older, current smoking prevalence was higher among smoking-related cancer survivors compared with non–smoking-related cancer survivors (19.78% vs 10.63%). After cancer diagnosis, the odds of continued cigarette smoking were twice as high among those with smoking-related cancers compared with those with non–smoking-related cancers.

**Meaning:**

Compared with non–smoking-related cancer survivors, smoking-related cancer survivors have a higher risk of being cigarette smokers and of continuing smoking.

## Teaser

This cross-sectional study examines cigarette smoking prevalence and behaviors (continuing or quitting smoking) among cancer survivors and compares them between survivors of smoking-related cancers and non–smoking-related cancers.

## Introduction

The cancer survivor population in the US has increased over the past half century to an estimated 18.1 million in 2020 and is projected to increase to more than 20 million by 2026.^1,2^ This phenomenon is largely due to more cancer diagnoses as a result of an aging population and improvements in early detection and treatment of cancer.^3,4^ Healthy lifestyle behaviors (eg, smoking cessation, physical activity, maintaining healthy weight, and consuming a healthy diet) may help improve quality of life among cancer survivors and prevent recurrent and subsequent cancers.^4^

Smoking remains the leading preventable cause of disease and death in the US. Although numerous antitobacco efforts have made substantial contributions to public health, cigarette smoking continues to claim half a million lives annually as a result of lung cancer and other tobacco-related diseases.^5,6,7^ Compared with individuals who have never smoked, cigarette smokers are at increased risk of having detrimental health conditions such as cancer (eg, leukemia and lung cancer), cardiovascular disorders (eg, atherosclerosis and hypertension), respiratory diseases (eg, chronic obstructive pulmonary disease, pneumonia, chronic bronchitis, and emphysema), diabetes, and oral conditions (eg, periodontal disease, oral leukoplakia, and oral cancer), among others.^8,9,10,11,12^ Consequently, smokers tend to be admitted to the hospital more often than nonsmokers and, ultimately, have a higher risk of premature death,^13^ which carries an associated economic cost to the nation of nearly $300 billion a year.^6,14,15^

Many cancer survivors continue to smoke, despite the knowledge that continued smoking leads to poor clinical outcomes and shorter survival times.^16,17,18^ It is established that individuals who continue to smoke after a cancer diagnosis are at increased risk of death from smoking-related cardiovascular and respiratory complications, as well as higher risk of cancer recurrence, the development of second primary cancers, and complications from treatment.^19,20,21^ Continuing to smoke after a cancer diagnosis has also been associated with overall poorer physical, social, and emotional functioning.^22,23^ Previous studies^24,25,26,27,28^ have examined the smoking practices among cancer survivors in the US. However, those studies did not include indicators of mental health, nor did they measure the impact on smoking behaviors of other tobacco-related nonmalignant conditions among cancer survivors who smoke.^24,25,26^ Furthermore, it remains unclear whether cigarette smoking behaviors vary between survivors of smoking-related cancers (SRCs) and non–smoking-related cancers (NSRCs).

In this cross-sectional study, we investigated the prevalence and patterns of continuing or quitting smoking after cancer diagnosis by taking into account key potential confounders, while comparing smoking behaviors between survivors of SRCs and NSRCs. Understanding the factors that contribute to smoking behaviors among cancer survivors will assist in the development of smoking cessation interventions targeted to and tailored for this high-risk group.

## Methods

### Data and Sampling Design

We used data from the 2017 National Health Interview Survey (NHIS), a cross-sectional household survey of the civilian, noninstitutionalized population that resides in the US. Participants in the 2017 NHIS study provided written informed consent before participation, and the survey was approved by the research ethics review board of the National Center for Health Statistics. Our cross-sectional study was limited to adult participants (ie, respondents aged ≥18 years). This secondary data analysis of the 2017 NHIS data was exempt from institutional review board approval because all data sets are publicly available, in accordance with 45 CFR §46. This study follows the American Association for Public Opinion Research (AAPOR) reporting guideline.

A detailed description of the NHIS sampling design is reported by Parsons et al.^29^ Briefly, the data collection process used a multistage stratified area probability design to recruit households and implemented the survey in face-to-face interviews. In the first stage of the sampling design, 319 primary sampling units were sampled from approximately 1700 geographically defined primary sampling units from all 50 states and the District of Columbia. In the second stage of the sampling design, area segments (geographically defined areas within a primary sampling unit) and permit segments (defined using housing units built after the 2000 US Census) were used to sample households within a primary sampling unit.

### Identification and Classification of Cancer Survivors

In the 2017 NHIS survey, questions about cancer survivorship were administered by all US states and territories and the District of Columbia. Respondents were asked whether they had ever been told by a physician, nurse, or other health care professional that they had cancer. If they answered yes, they were asked how many different types of cancer they had had, their age at first diagnosis, and the type(s) of cancer. If respondents reported more than 1 cancer, they were included in the analyses separately for each cancer reported. Respondents who had an unknown history of cancer or who did not answer the question were excluded from the analyses.

Cancer survivors were classified into 2 categories depending on whether tobacco was positively associated with the type of cancer they were diagnosed with, per the 2014 Surgeon General Report^6^: survivors of SRCs (eg, cancers of the bladder, blood, cervix, colon, esophagus, kidney, larynx or windpipe, leukemia, lung, liver, mouth, tongue, lip, pancreas, rectum, stomach, throat or pharynx, and uterus) and survivors of NSRCs (eg, cancers of the bone, brain, breast, gallbladder, lymphoma, melanoma, ovary, prostate, skin, soft tissue, testis, and thyroid). Respondents who reported a prior cancer diagnosis at any other site were included in the population of other NSRC survivors.

### Smoking Behavior

Using NHIS definitions, individuals who smoked 100 cigarettes or more in their lifetime and who smoked every day or on some days at the time of the survey were defined as current smokers. Individuals who smoked fewer than 100 cigarettes in their lifetime were defined as never smokers. Individuals who smoked 100 cigarettes or more in their lifetime but did not smoke at the time of the survey were defined as former smokers. The information on current smokers included the number of cigarettes smoked per day, whether they were daily or smoked only some days, and whether they had tried to quit smoking in the last 12 months. Those respondents who were current smokers and stopped smoking for more than 24 hours in the last 12 months because they were trying to quit were classified as having attempted to quit smoking in the past 12 months but still smoking.

### Other Measures

We assessed demographic characteristics and tobacco use among survivors of SRCs and NSRCs and respondents without cancer. The NHIS data included information such as age, sex, race/ethnicity, education level, working status, poverty status, marital status, body mass index, and a variety of diseases. In addition to cancer, other acute or chronic diseases that are linked to tobacco use were reported by respondents, including diabetes, coronary artery disease, chronic obstructive pulmonary diseases (eg, emphysema, chronic bronchitis, and asthma), hypertension, arthritis, gastric ulcer, hay fever, high cholesterol, angina pectoris, and sinusitis. Selected indicators of mental health included in the survey were “depression/anxiety/emotional problem,” “fatigue/tiredness/weakness,” and hopelessness. In the 2017 NHIS, survey respondents were asked, “The next questions ask about difficulties you may have doing certain activities because of a HEALTH PROBLEM. By ‘health problem’ we mean any physical, mental, or emotional problem or illness (not including pregnancy)*.*” Those who mentioned “depression/anxiety/emotional problem” as a health problem were considered as having this condition. “Fatigue/tiredness/weakness” was defined in the same way. Hopelessness was ascertained using the following questions: “During the past 30 days, how often do you feel hopeless?” and the possible answers included all of the time, most of the time, some of the time, a little of the time, and none of the time. Items assessing smokeless tobacco or electronic nicotine delivery device use were not included in this study.

### Statistical Analysis

Because the NHIS data are based on a multistage sampling design, analyses were performed using the sample weights that take into account nonresponse and post hoc observed variations in age, sex, and race compared with the US census data. To account for these weights, we used the R statistical software package *survey*^30^ version 3.6.1 (R Project for Statistical Computing) to analyze the data. We referred to the 2010 US census data to calculate the age-adjusted and cancer type–specific prevalence of smoking by stratifying the survey data into 4 age groups: 18 to 24 years, 25 to 44 years, 45 to 64 years, and 65 years or older.

We compared demographic characteristics and tobacco use reported by all adult NHIS respondents, respondents with SRCs, those with NSRCs, and individuals without cancer. We performed descriptive analyses in which we calculated the age-adjusted prevalence of smokers who quit smoking after cancer diagnosis. Two questions from the NHIS survey were used to assess whether a participant quit or continued smoking after cancer diagnosis: (1) their age at cancer diagnosis, and (2) the age at which they quit smoking. To identify factors associated with quitting smoking compared with continuing smoking among cancer survivors, we performed survey logistic regression analysis with the R command *svyglm* using the quasibinomial link function to adjust for sociodemographic factors (eg, age, sex, education level, marital status, and race), mental health indicators (depression, anxiety, emotional problem, fatigue, tiredness, weakness, and hopelessness), and other tobacco-related comorbidities (eg, hypertension, asthma, emphysema, diabetes, and arthritis). The logistic regression model used was based on the complex survey design, with inverse-probability sample weighting and design-based standard errors. To avoid warnings regarding noninteger number of success in the estimation process, we used the quasibinomial link function per the package *survey’s* instructions. Because variables considered to be potential confounders of smoking behaviors were all included in the final model, we did not conduct any variable selection approaches (eg, stepwise regression analysis). In this analysis, respondents’ age (in years), as well as the years survived after cancer diagnosis, were included in the model as continuous variables. The percentages presented in this report are age-adjusted and weighted. For all statistical analyses, the significance level was calculated with 2-sided *t* tests and defined as *P* ≤ .05. Data analysis was performed from June to October 2019.

## Results

### Cancer Survivor Characteristics

The 2017 NHIS included data on 26 742 adults (mean [SD] age, 50.97 [18.61] years; survey weighted sample size, 246 647 271 participants). Table 1 describes the sociodemographic, health-related, and behavioral characteristics of the study population. Overall, 14 646 NHIS participants were female (51.76% survey weighted), 13 373 (60.29%) were married, and 21 996 (88.24%) were living above the poverty level. Less than one-half of NHIS participants (10 407 participants [46.07%]) were aged 18 to 44 years, 18 621 (63.95%) were non-Hispanic white, 3244 (16.01%) were Hispanic, 2853 (11.83%) were non-Hispanic black, and 1316 (5.90%) were Asian. In this population, 3068 individuals (9.42%, survey weighted) had cancer. Of the individuals who had cancer, 589 (19.96%) were SRC survivors, 2297 (74.50%) were NSRC survivors, 168 (4.96%) were survivors of both SRC and NSRC, and the remaining 14 (0.58%) had missing information about the type of cancer. Four hundred forty-nine SRC survivors (54.08%) were women, compared with 1412 NSRC survivors (54.30%). Ninety-six SRC survivors (15.69%) and 151 NSRC survivors (7.99%) were younger than 45 years. Among cancer survivors, individuals with SRCs were younger when diagnosed with cancer (mean age, 49.35 years; range, 47.77-50.94 years) compared with NSRC survivors (mean age, 52.93 years; range, 52.16-53.71 years). Non-Hispanic white individuals accounted for 625 SRC survivors (79.83%) and 2133 NSRC survivors (84.47%) (Table 1). Respondents with SRCs were economically disadvantaged compared with NSRC survivors; 98 SRC survivors (11.02%) and 184 NSRC survivors (6.79%) were living below the poverty level. Survivors of SRCs were less educated compared with NSRC survivors; only 74 SRC survivors (10.25%) had obtained a postgraduate degree, compared with 390 NSRC survivors (16.92%). Higher percentages of SRC survivors reported depression, anxiety, and emotional problems (35 survivors [8.01%] vs 73 survivors [4.51%]) and hopelessness (178 survivors [20.15%] vs 402 survivors [12.46%]), compared with NSRC survivors (Table 1).

**Table 1.** Estimated Sampling Weight–Adjusted Percentages of Individuals by Select Characteristics in the 2017 US National Health Interview Survey Study Population, the Population With Smoking Related Cancers, the Population With Non–Smoking-Related Cancers, and the Population Without Cancer

Variable	Weighted % (95% CI)			
	National Health Interview Survey population	Smoking-related cancer	Non–smoking-related cancer	No cancer
Participants, No. (%)	26 742 (100.0)	757 (2.35)	2465 (7.49)	23 520 (90.58)
Have cancer				
No	90.58 (90.13-91.01)^a^	0	0	100.00 (100.00-100.00)
Yes	9.42 (8.99-9.87)	100.00 (100.00-100.00)	100.00 (100.00-100.00)	0
Smoking-related cancers (among participants with cancer)				
No	75.08 (73.29-76.79)	0	93.75 (92.63-94.71)	NA
Yes	24.92 (23.21-26.71)	100.00 (100.00-100.00)	6.25 (5.29-7.37)^a^	NA
Non–smoking-related cancers (among participants with cancer)				
No	20.53 (18.91-22.26)	80.08 (76.74-83.05)	0	NA
Yes	79.47 (77.74-81.09)	19.92 (16.95-23.26)^a^	100.00 (100.00-100.00)	NA
When did you successfully quit smoking after cancer diagnosis?				
Within 2 y after cancer diagnosis	13.18 (10.53-16.38)	15.27 (10.84-21.10)	12.50 (9.43-16.39)	NA
After 2 y but no more than 5 y	8.28 (6.15-11.06)	4.15 (2.09-8.07)	10.03 (7.34-13.57)	NA
After 5 y but no more than 10 y	6.87 (4.71-9.92)	5.91 (2.99-11.38)	6.83 (4.37-10.54)	NA
After 10 y but no more than 20 y	10.96 (8.43-14.12)	7.60 (4.61-12.29)	13.44 (10.12-17.63)	NA
After 20 y but no more than 100 y	8.93 (6.76-11.69)	10.05 (6.39-15.46)	8.54 (6.14-11.78)	NA
Never	51.79 (47.21-56.34)	57.01 (49.60-64.11)	48.65 (43.28-54.06)	NA
Age range, y				
18-24	11.93 (11.23-12.66)	0.85 (0.36-1.99)	0.38 (0.17-0.88)	13.12 (12.37-13.92)
25-44	34.14 (33.35-34.94)	14.84 (11.86-18.42)	7.61 (6.39-9.05)	36.72 (35.87-37.58)
45-64	33.85 (33.06-34.65)	33.61 (29.40-38.10)	35.66 (33.42-37.97)	33.65 (32.80-34.51)
65-86	20.08 (19.44-20.74)	50.69 (46.13-55.24)	56.34 (53.93-58.72)	16.51 (15.90-17.13)
Age at first cancer diagnosis, mean (range), y	52.09 (51.38-52.81)	49.35 (47.77-50.94)	52.93 (52.16-53.71)	NA
Sex				
Female	51.76 (51.00-52.52)	54.08 (49.84-58.27)	54.30 (51.80-56.78)	51.50 (50.69-52.31)
Male	48.24 (47.48-49.00)	45.92 (41.73-50.16)	45.70 (43.22-48.20)	48.50 (47.69-49.31)
Race/ethnicity				
Non-Hispanic white	63.95 (62.24-65.63)	79.83 (75.51-83.55)	84.47 (82.11-86.56)	61.91 (60.16-63.63)
Hispanic	16.01 (14.65-17.47)	6.90 (4.75-9.93)	5.83 (4.65-7.29)	17.05 (15.62-18.58)
Non-Hispanic Asian	5.90 (5.29-6.58)	4.07 (2.54-6.45)	2.47 (1.56-3.91)	6.21 (5.57-6.92)
Non-Hispanic black	11.83 (10.85-12.89)	7.17 (5.22-9.75)	5.62 (4.45-7.08)	12.45 (11.43-13.54)
Other	2.31 (1.91-2.79)	2.04 (1.07-3.84)	1.61 (1.09-2.36)	2.38 (1.96-2.89)
Marital status				
Never married	22.57 (21.73-23.42)	6.79 (5.00-9.15)	6.29 (5.40-7.32)	24.24 (23.36-25.14)
Married	60.29 (59.42-61.15)	65.66 (61.76-69.36)	66.91 (64.71-69.04)	59.60 (58.68-60.51)
Single after marriage	17.15 (16.62-17.68)	27.55 (24.27-31.10)	26.80 (24.83-28.86)	16.16 (15.62-16.71)
Working status				
Not working	31.26 (30.40-32.13)	57.21 (52.82-61.48)	57.73 (55.26-60.16)	28.56 (27.71-29.43)
Working	68.74 (67.87-69.60)	42.79 (38.52-47.18)	42.27 (39.84-44.74)	71.44 (70.57-72.29)
Education level				
High school graduate or general equivalency diploma	24.21 (23.41-25.03)	25.39 (21.80-29.34)	22.46 (20.53-24.51)	24.33 (23.48-25.20)
No high school diploma	11.85 (11.16-12.56)	12.17 (9.58-15.33)	9.92 (8.57-11.45)	11.98 (11.28-12.73)
Some college or associate’s degree	30.33 (29.39-31.28)	33.25 (29.30-37.46)	29.46 (27.32-31.70)	30.34 (29.35-31.34)
Undergraduate degree	21.20 (20.40-22.02)	18.94 (15.64-22.75)	21.24 (19.23-23.40)	21.23 (20.40-22.08)
Postgraduate degree	12.42 (11.73-13.13)	10.25 (7.75-13.44)	16.92 (15.11-18.91)	12.12 (11.41-12.86)
Poverty level				
Above poverty level	88.24 (87.51-88.94)	88.98 (85.85-91.48)	93.21 (91.76-94.42)	87.83 (87.06-88.56)
Below poverty level	11.76 (11.06-12.49)	11.02 (8.52-14.15)	6.79 (5.58-8.24)	12.17 (11.44-12.94)
Alcohol use status				
Never	19.74 (18.73-20.78)	14.93 (12.05-18.35)	15.41 (13.60-17.42)	20.21 (19.15-21.30)
Former	13.44 (12.88-14.02)	22.82 (19.33-26.73)	19.49 (17.68-21.42)	12.74 (12.15-13.35)
Current	66.83 (65.78-67.86)	62.25 (57.61-66.67)	65.10 (62.62-67.51)	67.06 (65.96-68.14)
Hypertension				
No	69.36 (68.59-70.12)	43.90 (39.47-48.43)	46.53 (44.04-49.03)	71.75 (70.99-72.51)
Yes	30.64 (29.88-31.41)	56.10 (51.57-60.53)	53.47 (50.97-55.96)	28.25 (27.49-29.01)
High cholesterol				
No	71.26 (70.44-72.06)	52.46 (47.98-56.90)	50.14 (47.77-52.52)	73.40 (72.57-74.21)
Yes	28.74 (27.94-29.56)	47.54 (43.10-52.02)	49.86 (47.48-52.23)	26.60 (25.79-27.43)
Coronary artery disease				
No	95.63 (95.32-95.91)	86.93 (83.27-89.89)	88.42 (86.75-89.91)	96.39 (96.11-96.65)
Yes	4.37 (4.09-4.68)	13.07 (10.11-16.73)	11.58 (10.09-13.25)	3.61 (3.35-3.89)
Angina pectoris				
No	98.21 (98.02-98.38)	94.74 (92.50-96.34)	95.63 (94.60-96.47)	98.48 (98.30-98.64)
Yes	1.79 (1.62-1.98)	5.26 (3.66-7.50)	4.37 (3.53-5.40)	1.52 (1.36-1.70)
Heart attack				
No	96.92 (96.68-97.15)	89.68 (86.88-91.94)	93.47 (92.32-94.47)	97.37 (97.13-97.59)
Yes	3.08 (2.85-3.32)	10.32 (8.06-13.12)	6.53 (5.53-7.68)	2.63 (2.41-2.87)
Heart disease				
No	92.28 (91.84-92.71)	84.93 (81.37-87.92)	82.33 (80.48-84.03)	93.22 (92.78-93.64)
Yes	7.72 (7.29-8.16)	15.07 (12.08-18.63)	17.67 (15.97-19.52)	6.78 (6.36-7.22)
Stroke				
No	96.85 (96.59-97.10)	91.32 (88.72-93.37)	92.28 (90.90-93.46)	97.34 (97.08-97.58)
Yes	3.15 (2.90-3.41)	8.68 (6.63-11.28)	7.72 (6.54-9.10)	2.66 (2.42-2.92)
Chronic obstructive pulmonary disease				
No	96.64 (96.34-96.91)	87.08 (84.02-89.62)	92.49 (91.20-93.61)	97.15 (96.86-97.42)
Yes	3.36 (3.09-3.66)	12.92 (10.38-15.98)	7.51 (6.39-8.80)	2.85 (2.58-3.14)
Emphysema				
No	98.61 (98.44-98.77)	93.14 (90.68-94.99)	97.40 (96.59-98.02)	98.83 (98.67-98.98)
Yes	1.39 (1.23-1.56)	6.86 (5.01-9.32)	2.60 (1.98-3.41)	1.17 (1.02-1.33)
Chronic bronchitis				
No	96.52 (96.26-96.77)	91.83 (89.47-93.70)	94.36 (93.27-95.28)	96.79 (96.52-97.04)
Yes	3.48 (3.23-3.74)	8.17 (6.30-10.53)	5.64 (4.72-6.73)	3.21 (2.96-3.48)
Asthma				
No	86.58 (86.02-87.11)	81.15 (77.57-84.27)	85.19 (83.49-86.74)	86.80 (86.20-87.37)
Yes	13.42 (12.89-13.98)	18.85 (15.73-22.43)	14.81 (13.26-16.51)	13.20 (12.63-13.80)
Gastric ulcer				
No	93.81 (93.43-94.17)	85.99 (82.88-88.61)	88.21 (86.52-89.71)	94.43 (94.05-94.78)
Yes	6.19 (5.83-6.57)	14.01 (11.39-17.12)	11.79 (10.29-13.48)	5.57 (5.22-5.95)
Diabetes				
No	87.76 (87.24-88.27)	77.74 (73.72-81.30)	80.02 (78.12-81.79)	88.61 (88.07-89.13)
Yes	12.24 (11.73-12.76)	22.26 (18.70-26.28)	19.98 (18.21-21.88)	11.39 (10.87-11.93)
Hay fever				
No	91.93 (91.39-92.44)	89.34 (86.68-91.52)	87.36 (85.55-88.98)	92.37 (91.82-92.89)
Yes	8.07 (7.56-8.61)	10.66 (8.48-13.32)	12.64 (11.02-14.45)	7.63 (7.11-8.18)
Sinusitis				
No	87.48 (86.93-88.02)	82.53 (79.26-85.38)	80.89 (79.05-82.60)	88.13 (87.57-88.67)
Yes	12.52 (11.98-13.07)	17.47 (14.62-20.74)	19.11 (17.40-20.95)	11.87 (11.33-12.43)
Arthritis				
No	76.25 (75.52-76.97)	51.51 (46.79-56.20)	50.63 (48.20-53.06)	78.83 (78.12-79.53)
Yes	23.75 (23.03-24.48)	48.49 (43.80-53.21)	49.37 (46.94-51.80)	21.17 (20.47-21.88)
Depression, anxiety, or emotional problem				
No	93.07 (92.32-93.75)	91.99 (88.24-94.61)	95.49 (94.15-96.53)	92.79 (91.95-93.56)
Yes	6.93 (6.25-7.68)	8.01 (5.39-11.76)	4.51 (3.47-5.85)	7.21 (6.44-8.05)
Fatigue, tiredness, or weakness				
No	98.85 (98.57-99.08)	99.32 (98.22-99.74)	99.21 (98.61-99.56)	98.77 (98.44-99.03)
Yes	1.15 (0.92-1.43)	0.68 (0.26-1.78)	0.79 (0.44-1.39)	1.23 (0.97-1.56)
Hopelessness				
None of the time	86.50 (85.88-87.10)	79.85 (76.09-83.16)	87.54 (85.79-89.10)	86.56 (85.90-87.19)
All of the time	0.89 (0.76-1.04)	1.59 (0.83-3.02)	0.82 (0.52-1.29)	0.88 (0.74-1.03)
Most of the time	1.22 (1.07-1.38)	2.00 (1.20-3.30)	1.34 (0.84-2.14)	1.18 (1.04-1.35)
Some of the time	4.94 (4.61-5.30)	8.67 (6.42-11.62)	4.26 (3.46-5.25)	4.92 (4.56-5.30)
A little of the time	6.44 (6.05-6.87)	7.88 (5.88-10.48)	6.04 (4.87-7.45)	6.47 (6.03-6.93)

Abbreviation: NA, not applicable.

^a^ The overlap observed here is because patients with more than 1 cancer were included separately in the analyses for each cancer diagnosed.

Considering nonmalignant comorbidities associated with tobacco use, SRC survivors were more frequently diagnosed with chronic obstructive pulmonary diseases (107 survivors [12.92%] vs 205 survivors [7.51%]) and gastric ulcer (112 survivors [14.01%] vs 301 survivors [11.79%]) compared with NSRC survivors. Of respondents with SRCs and NSRCs, 36 (15.27%) and 55 (12.50%) survivors, respectively, successfully quit cigarette smoking within the first 2 years after the cancer diagnosis (Table 1).

### Prevalence and Patterns of Smoking Among Cancer Survivors

The age-adjusted prevalence of current, former, and never smokers among cancer survivors and by cancer type is reported in Table 2. Overall, 372 cancer survivors (13.16%) were current smokers. The proportion of current smokers was higher among SRC survivors (145 survivors [19.78%]) compared with NSRC survivors (251 survivors [10.63%]) and individuals without cancer (3637 individuals [14.20%]). Among SRCs, the proportion of current smokers was lower for lung cancer (15 survivors [10.19%]), compared with kidney (17 survivors [30.08%]), cervix (56 survivors [32.24%]), and pancreas (2 survivors [41.06%]) cancers. Among NSRCs, the proportion of current smokers was lower for lymphoma (4 survivors [1.68%]), compared with breast (51 survivors [12.64%]), brain (6 survivors [16.18%]), and ovary (13 survivors [26.32%]) cancers.

**Table 2.** Estimated Age-Adjusted Prevalence of Current Smokers, Former Smokers, and Never Smokers Among US Adults Aged 18 Years or Older by Cancer Type: US National Health Interview Survey, 2017

Variable	Participants		Percentage (95%CI)		
	Observed No.	Survey-weighted No.	Current smokers^a^	Former smokers^b^	Never smokers^c^
Population	26 742	246 657 271	14.12 (13.51-14.75)	21.73 (21.06-22.41)	64.15 (63.23-65.05)
Participants with cancer	3068	23 222 976	13.16 (10.99-15.67)	26.13 (23.2-29.29)	60.71 (57.01-64.3)
Participants without cancer	23 655	223 305 854	14.20 (13.57-14.85)	21.02 (20.32-21.73)	64.79 (63.85-65.71)
Smoking-related cancers^d^	757	5 787 453	19.78 (15.62-24.72)	29.11 (23.66-35.24)	51.11 (44.26-57.91)
Non–smoking-related cancers	2465	18 454 422	10.63 (8.477-13.26)	24.26 (21.26-27.53)	65.11 (61.44-68.61)
Bladder^e^	77	587 574	16.65 (8.396-30.33)	29.72 (19.51-42.44)	53.64 (42.5-64.42)
Blood^e^	22	149 637	21.24 (1.684-80.95)	48.28 (9.712-89.01)	30.48 (9.508-64.65)
Bone	24	174 174	3.12 (0.11-48.44)	54.57 (20.38-84.93)	42.31 (13.53-77.47)
Brain	21	202 250	16.18 (4.172-46.12)	27.15 (11.63-51.36)	56.67 (39.36-72.48)
Breast	563	3 957 798	12.64 (5.961-24.82)	23.27 (17.96-29.58)	64.10 (53.54-73.44)
Cervix^e^	173	1 219 324	32.24 (23.77-42.06)	30.76 (22.33-40.72)	37.00 (28.27-46.67)
Colon^e^	170	1 350 653	7.65 (3.67-15.29)	23.46 (14.48-35.68)	68.89 (55.86-79.48)
Esophagus^e^	10	74 214	2.48 (NE)	25.18 (NE)	72.34 (NE)
Gallbladder	0	0	NE	NE	NE
Kidney^e^	76	556 126	30.08 (12.88-55.6)	17.37 (11.8-24.83)	52.55 (30.73-73.43)
Larynx^e^	12	121 505	0.00 (NE)	90.12 (NE)	9.88 (NE)
Leukemia^e^	45	392 573	10.64 (3.10-30.74)	23.43 (10.54-44.27)	65.94 (46.11-81.41)
Liver^e^	14	80 249	18.75 (NE)	22.55 (NE)	58.70 (NE)
Lung^e^	97	634 886	10.19 (4.57-21.19)	34.43 (24.91-45.39)	55.38 (46.63-63.8)
Lymphoma	94	694 268	1.68 (0.59-4.67)	21.18 (14.05-30.64)	77.14 (67.71-84.45)
Melanoma	245	1 880 127	15.00 (7.64-27.34)	32.08 (24.69-40.48)	52.92 (42.12-63.46)
Mouth^e^	15	134 983	17.73 (3.25-58.03)	32.07 (10.63-65.22)	50.19 (29.86-70.46)
Ovary	58	496 080	26.32 (13.53-44.92)	20.67 (11.11-35.2)	53.01 (35.77-69.56)
Pancreas^e^	18	137 481	41.06 (NE)	25.69 (NE)	33.25 (NE)
Prostate	369	2 813 128	9.30 (5.88-14.41)	22.62 (17.65-28.51)	68.07 (61.88-73.69)
Rectum^e^	22	167 961	8.08 (1.29-37.2)	32.48 (13.55-59.63)	59.44 (37.9-77.87)
Skin (nonmelanoma)	653	4 899 662	4.96 (3.42-7.13)	28.06 (21.32-35.96)	66.98 (59.09-74.02)
Skin (type unknown)	288	2 058 736	11.22 (5.40-21.89)	31.14 (21.56-42.67)	57.64 (46.41-68.13)
Soft tissue	16	126 321	9.44 (2.0-34.77)	9.71 (1.83-38.33)	80.85 (54.86-93.62)
Stomach^e^	29	319 306	24.23 (6.662-58.9)	31.52 (11.37-62.29)	44.25 (18.56-73.44)
Testis	19	153 770	10.94 (1.47-50.27)	31.58 (14.45-55.78)	57.48 (33.66-78.27)
Throat^e^	22	169 245	7.74 (0.26-72.71)	25.30 (5.92-64.6)	66.95 (29.22-90.86)
Thyroid	79	631 050	5.33 (2.49-11.03)	15.02 (8.84-24.38)	79.65 (69.69-86.95)
Uterus	125	888 225	19.13 (10.27-32.84)	16.20 (9.48-26.31)	64.66 (50.32-76.78)
Other	168	1 289 535	18.74 (9.57-33.47)	32.52 (22.70-44.17)	48.73 (35.76-61.88)

Abbreviation: NE, not estimable because of low sample size.

^a^ Current smokers are persons who reported smoking at least 100 cigarettes during their lifetime and who, at the time of the interview, reported smoking every day or some days.

^b^ Former smokers are persons who reported smoking at least 100 cigarettes during their lifetime but who currently did not smoke.

^c^ Never smokers are persons who reported never smoking 100 cigarettes during their lifetime.

^d^ Includes cancers of the bladder, blood, cervix, colon, esophagus, kidney, larynx, windpipe, leukemia, lung, liver, mouth, tongue, lip, pancreas, rectum, stomach, throat, pharynx, and uterus.

^e^ Smoking-related cancers.

The age-adjusted prevalence of daily smokers and those who smoked some days, as well as the mean number of cigarettes smoked per day by cancer type among current smokers, are reported in eTable 1 and eTable 2 in the Supplement, respectively. Of note, the percentage of daily smokers was similar among survivors of SRCs and NSRCs (121 survivors [74.83%] vs 205 survivors [79.89%]) and the number of cigarettes smoked per day among daily smokers was virtually identical (SRCs, 13.82 cigarettes per day; NSRCs, 13.32 cigarettes per day).

### Percentage of Smokers Who Quit Smoking or Attempted to Quit After Cancer Diagnosis

The age-adjusted percentage of smokers who quit smoking after cancer diagnosis and that of cancer survivors who attempted to quit within the past 12 months but were still smoking at the time of the survey are reported in Table 3. Overall, 309 (43.96%) survivors who previously smoked quit after cancer diagnosis; this proportion was lower in individuals with SRCs (104 individuals [40.13%]) compared with NSRC survivors (233 individuals [48.36%]). Overall, 372 survivors (56.04%) reported continuing smoking. At the time of the survey, 15 lung cancer survivors (54.60%) and 51 breast cancer survivors (70.59%) continued to smoke. Notably, 176 of cancer survivors (56.49%) who continued to smoke after cancer diagnosis attempted but failed to quit smoking within the last 12 months. This proportion was lower among survivors of NSRCs (115 survivors [47.00%]) compared with survivors of SRCs (72 survivors [58.95%]).

**Table 3.** Age-Adjusted Prevalence of Smokers Who Quit After Diagnosis of Cancer Among Individuals Who Were Current Smokers at the Time of Diagnosis of Cancer and Individuals Who Tried to Quit Smoking in the Past 12 Months Among Individuals Who Are Still Smoking After Diagnosis of Cancer, by Cancer Type: US National Health Interview Survey, 2017

Cancer type	Participants who quit smoking after cancer diagnosis			Participants who attempted to quit smoking in the past 12 mos but are still smoking		
	Observed No.	Survey-weighted No.	Percentage (95% CI)	Observed No.	Survey-weighted No.	Percentage (95% CI)
Participants with cancer	681	4 969 522	43.96 (36.05-52.19)	372	2 573 558	56.49 (49.59-63.15)
Smoking-related cancers	249	1 717 138	40.13 (29.91-51.29)	145	978 888	58.95 (49.76-67.55)
Non–smoking-related cancers	484	3 572 850	48.36 (40.94-55.84)	251	1 738 353	47.00 (35.95-58.36)
Bladder^a^	27	195 879	42.58 (12.52-79.35)	15	83 946	54.32 (NE)
Blood^a^	3	22 203	19.75 (NE)	2	10 553	0.00 (NE)
Bone	4	31 065	87.32 (NE)	1	3938	NE
Brain	12	96 570	52.91 (13.84-88.72)	6	47 412	40.75 (NE)
Breast	99	713 138	29.41 (21.02-39.46)	51	358 935	30.90 (19.88-44.63)
Cervix^a^	95	658 578	39.54 (26.64-54.07)	56	407 523	49.77 (40.15-59.4)
Colon^a^	29	185 904	52.84 (26.28-77.89)	17	99 414	44.18 (25.06-65.2)
Esophagus^a^	1	3537	0.00 (NE)	1	3537	0.00 (NE)
Gallbladder	0	0	NE	0	0	NE
Kidney^a^	26	172 101	13.84 (5.751-29.73)	17	126 170	41.04 (NE)
Larynx^a^	4	35 692	100.00 (NE)	0	0	NE
Leukemia^a^	10	78 724	43.11 (5.80-90.32)	6	42 537	21.18 (NE)
Liver^a^	5	20 409	0.00 (NE)	5	20 409	13.48 (NE)
Lung^a^	36	207 937	45.4 (20.98-72.25)	15	85 594	33.63 (2.021-92.57)
Lymphoma	12	75 559	84.97 (NE)	4	18 207	49.09 (NE)
Melanoma	49	416 368	52.13 (35.28-68.51)	17	131 222	32.16 (3.911-84.66)
Mouth^a^	4	46 775	38.92 (NE)	3	28 571	100.00 (NE)
Ovary	21	151 427	32.17 (16.64-52.99)	13	895 68	51.15 (9.812-90.97)
Pancreas^a^	2	11 552	0.00 (NE)	2	11 552	66.93 (NE)
Prostate	77	623 424	41.57 (22.91-63.01)	42	308 265	35.87 (16.96-60.49)
Rectum^a^	4	21 943	12.93 (NE)	3	18 573	33.07 (NE)
Skin (nonmelanoma)	120	843 506	64.43 (50.87-76.02)	50	333 628	32.22 (22.63-43.58)
Skin (type unknown)	57	381 876	47.11 (23.46-72.13)	29	173 454	41.94 (13.91-76.34)
Soft tissue	3	17 570	0.00 (NE)	3	17 570	0.00 (NE)
Stomach^a^	11	942 66	44.17 (NE)	6	49 622	80.25 (NE)
Testis	5	50 676	46.54 (NE)	2	28 620	68.79 (NE)
Throat^a^	8	50 652	65.22 (NE)	2	17 917	33.07 (NE)
Thyroid	12	70 766	32.64 (11.43-64.53)	8	35 964	18.44 (0.66-88.44)
Uterus	27	187 120	18.17 (7.20-38.86)	20	139 346	65.20 (23.90-91.79)
Other	44	337 629	46.97 (26.56-68.44)	22	1 60 640	46.66 (13.19-83.43)

Abbreviation: NE, not estimable because of low sample size

^a^ Smoking-related cancers.

### Factors Associated With Continuing Cigarette Smoking Compared With Quitting After Cancer Diagnosis

Factors associated with continuing smoking compared with quitting smoking after cancer diagnosis are presented in Table 4. After cancer diagnosis, SRC survivors had greater odds of continued smoking compared with NSRC survivors (odds ratio [OR], 2.10; 95% CI, 1.12-3.93; *P* = .02). Older cancer survivors had lower odds of continued smoking compared with younger cancer survivors (OR, 0.95; 95% CI, 0.93-0.97; *P* = .001). Men had higher odds of continued smoking compared with women (OR, 1.93; 95% CI, 1.05-3.57; *P* = .04), and Hispanic participants had lower odds of continued smoking compared with non-Hispanic white participants (OR, 0.18; 95% CI, 0.05-0.68; *P* = .01). Married individuals had lower odds than those never married of continued smoking after cancer diagnosis (OR, 0.33; 95% CI, 0.12-0.96; *P* = .04). Cancer survivors with asthma (OR, 0.35; 95% CI, 0.17-0.72; *P* = .005) and coronary artery disease (OR, 0.30; 95% CI, 0.11-0.79; *P* = .02) had lower odds of continued cigarette smoking after cancer diagnosis, whereas those with angina pectoris (OR, 5.40; 95% CI, 1.33-21.91; *P* = .02) and chronic bronchitis (2.55; 95% CI, 1.05-6.19; *P* = .04) had higher odds of continued smoking.

**Table 4.** Survey Logistic Regression Analysis of Smokers Who Continue to Smoke After Diagnosis of Cancer vs Those Who Quit After the Diagnosis of Cancer: US National Health Interview Survey, 2017^a^

Characteristic	OR (95% CI)	*P* value
Survivors of smoking-related cancers (vs non–smoking-related cancers)	2.10 (1.12-3.93)	.02
Duration of survival after cancer diagnosis, y	0.95 (0.93-0.97)	<.001
Age, y	0.95 (0.92-0.98)	.001
Male (vs female)	1.93 (1.05-3.57)	.04
Race/ethnicity		
Hispanic (vs non-Hispanic white)	0.18 (0.05-0.68)	.01
Other (vs non-Hispanic white)	2.07 (0.73-5.89)	.17
Non-Hispanic black (vs non-Hispanic white)	0.54 (0.2-1.44)	.22
Marital status		
Married (vs never married)	0.33 (0.12-0.96)	.04
Widowed, separated, or divorced (vs never married)	0.94 (0.32-2.79)	.91
Work status, working (vs not working)	0.81 (0.42-1.53)	.51
Education		
No diploma (vs high school)	1.83 (0.82-4.11)	.14
Some college (vs high school)	0.74 (0.36-1.53)	.42
Undergraduate (vs high school)	0.47 (0.19-1.16)	.11
Postgraduate (vs high school)	0.48 (0.16-1.44)	.19
Body mass index		
Underweight (vs normal weight)	5.63 (1.03-30.68)	.05
Overweight (vs normal weight)	1.49 (0.71-3.13)	.29
Obese (vs normal weight)	1.10 (0.53-2.27)	.81
Poverty status, below poverty level (vs above)	1.24 (0.57-2.68)	.59
Alcohol use status, ever (vs never)	0.23 (0.10-0.54)	.001
Comorbid conditions		
Hypertension	0.86 (0.46-1.63)	.65
High cholesterol	0.70 (0.37-1.32)	.27
Coronary artery disease	0.30 (0.11-0.79)	.02
Angina pectoris	5.40 (1.33-21.91)	.02
Stroke	2.03 (0.84-4.86)	.12
Emphysema	0.72 (0.32-1.62)	.43
Asthma	0.35 (0.17-0.72)	.005
Ulcer	1.87 (0.94-3.71)	.08
Diabetes	0.64 (0.33-1.26)	.20
Hay fever	0.93 (0.41-2.12)	.86
Sinusitis	1.16 (0.59-2.31)	.67
Chronic bronchitis	2.55 (1.05-6.19)	.04
Arthritis	1.04 (0.58-1.88)	.89
Depression, anxiety, or emotional problems	0.33 (0.11-1.01)	.05
Fatigue, tiredness, or weakness	3.02 (0.42-21.46)	.27
Experienced hopelessness		
All the time (vs none)	8.18 (0.67-100.26)	.10
Most of the time (vs none)	1.77 (0.25-12.76)	.57
Sometimes (vs none)	1.82 (0.70-4.72)	.22
Little of the time (vs none)	0.82 (0.28-2.41)	.72

Abbreviation: OR, odds ratio.

^a^ There were 372 smokers who continued to smoke after diagnosis of cancer and 309 who quit after the diagnosis of cancer.

## Discussion

In this study, we assessed smoking behaviors among US adult cancer survivors using the 2017 NHIS data. We found the prevalence of cigarette smoking in this high-risk group (13.16%) to be similar to that of the US general adult population (13.7%)^31^ despite compelling evidence supporting the adverse consequences of smoking on health and survival after cancer diagnosis.^32,33^ Current smoking prevalence was substantially higher among SRC survivors (19.78%) compared with NSRC survivors (10.63%) and individuals without cancer (14.20%). In a previous analysis of the 2009 Behavioral Risk Factor Surveillance System data, the prevalence of smoking was also found to be higher among cancer survivors with SRCs (27%) compared with NSRC survivors (16%) and individuals without cancer (18%).^26^ The lower rates observed in our study could be attributed to the recency of our data, the use of an updated classification of SRCs (the 2014 US Surgeon General report), and the use of a different sampling approach to enroll study participants.

Compared with NSRC survivors, a larger proportion of SRC survivors was younger than 45 years, less educated, and had incomes below the poverty level. According to existing smoking data among the US population, these characteristics are associated with high smoking prevalence.^34^ Smoking rates are highest among young people and decline with increasing age.^34^ Furthermore, the higher smoking prevalence among SRC survivors (compared with NSRC survivors) may also be driven by lower income and lesser educated populations, which have significantly higher smoking rates in the general population.^34^ Socioeconomic factors such as lower level of education and lower income may have influenced our findings, because they have been linked with high smoking rates, specifically among cancer survivors.^35^

A key finding of this study is that individuals with SRCs had lower odds of quitting smoking after cancer diagnosis. However, the existing literature is inconsistent. The odds of quitting smoking after cancer diagnosis were found to be higher among SRC survivors (and not NSRC survivors) in a previous study.^36^ On the other hand, a recent report^37^ from a comprehensive smoking cessation intervention in an oncology setting found that cessation rates did not differ between those with and without SRCs. Our study accounted for the presence of multiple tobacco-related comorbidities. This is reflected in the higher odds of continued smoking among cancer survivors with comorbidities like angina pectoris and chronic bronchitis.

It was tempting to speculate that there would be greater nicotine dependence among those with SRCs compared with NSRCs, but no precancer diagnosis level of smoking dependence data were available to assess this hypothesis. Furthermore, eTable 1 and eTable 2 in the Supplement show that the percentage of daily smokers was similar among SRCs and NSRCs (74.83% vs 79.89%) and the number of cigarettes smoked per day among daily smokers was virtually identical (13.82 cigarettes per day for SRCs vs 13.32 cigarettes per day for NSRCs). Smokers may have cut down after diagnosis, but those data were not available. Therefore, although one would expect greater motivation to quit among SRC survivors (ie, motivated by realizing one’s smoking likely contributed to their cancer), the NHIS data did not suggest higher dependence on nicotine among survivors of SRCs.

There was substantial interest in smoking cessation, because 56.49% of cancer survivors who smoked attempted to quit, unsuccessfully, in the past year, whereas 43.96% of cancer survivors who were smokers at the time of cancer diagnosis subsequently quit cigarette smoking. This is higher than the 49% rate among cancer survivors who reported making a past year quit attempt in the 2015 NHIS,^24^ reflecting a growing influence of tobacco cessation advocacy and efforts among adult cancer survivors in the US. Considering that a high proportion of quit attempts among US adult cancer survivors are unsuccessful, improvement and integration of smoking cessation into oncology care may help increase interest in and success of quitting smoking among in this high-risk population.^37,38,39^ Although a large majority of oncology practitioners ask about tobacco use, they may be less likely to be involved in discussing treatment options.^40,41,42^

In this study, older people as well as individuals with asthma or coronary artery disease had lower odds of continuing smoking after cancer diagnosis. Consistent with our results, a study on cancer survivorship found that a substantial proportion of cancer survivors also has heart disease, diabetes, asthma, and other health-related illness.^43^ Persons with comorbidities are typically older and have more interaction with health care practitioners and, therefore, more opportunity for counseling to quit smoking. Thus, the lower smoking prevalence among the older population of cancer survivors may be partly explained by comorbidities. However, these results should be interpreted with caution, considering the higher survival rates observed among cancer survivors who are nonsmokers, compared with those who are smokers.^6,7^ This was notably evidenced among never-smoking lung cancer survivors in whom lung cancer harbors targetable long survival variants like *EGFR*.^44,45^

Interestingly, within the group of SRC survivors, those with lung cancer had a lower rate of smoking (10.19%) compared to individuals with other types of cancer (eg, 30.08% kidney) which could be due to the lethality of lung cancer, especially for continuing smokers. Also, according to self-reported knowledge of SRC among patients visiting a urology clinic,^46^ it has been observed that many patients are not aware that smoking is a critical risk factor for kidney cancer as well. This could be another explanation as to why kidney cancer survivors continue to smoke at higher rates. Therefore, immediate actions aimed at raising awareness about the association of smoking with certain cancer types and educating patients about the consequences of smoking after cancer treatment and survival are warranted.

### Strengths and Limitations

Our study had strengths. First, we included cancers reported in the NHIS and classified them as SRCs and NSRCs per the 2014 Surgeon General report, unlike previous studies that have focused on state-level data of a limited number of SRCs and NSRCs^25^ or used an older classification of SRCs.^26^ To the best of our knowledge, this is the first nationwide study to comprehensively examine the prevalence and determinants of smoking behaviors in the US adult population of cancer survivors, since the 2014 Surgeon General report was issued. Second, beyond demographic characteristics, data about other comorbidities were included in our study.

The study limitations included the cross-sectional nature of the data, which could be affected by a number of biases, including the low–response rate bias and social desirability bias. Because mortality is higher among cancer survivors who continue to smoke,^6,7^ this group of patients is less likely to survive than survivors of NSRCs and might be underrepresented in our data (ie, survival bias). Similarly, the prevalence of cancer survivors who quit cigarette smoking after diagnosis may be overestimated, because patients with cancer who do not quit smoking are more likely to die sooner.^6,7^ Therefore, both the causality and directionality of the associations observed in this study could not be established. Furthermore, the data on smoking behaviors, health conditions, and other risk factors were self-reported.

## Conclusions

Given what is known about the adverse effects of continued smoking after cancer diagnosis,^6^ survivors of any cancer, particularly SRCs, are at elevated risk for developing further disease and should be prime targets for intervention.^47,48,49,50^ These findings reinforce the importance of broad smoking cessation efforts among cancer survivors.^51,52,53^ In the US adult population of cancer survivors, current smoking prevalence continues to be higher among SRC survivors compared with NSRC survivors. Although smoking cessation interventions are critically important for all cancer survivors, special efforts should target survivors of SRCs.

## Notes

Supplement. **eTable 1. **Age-Adjusted Prevalence of Daily and Someday Smokers Among Individuals Who Were Current Smokers by Cancer Type: National Health Interview Survey, United States, 2017 **eTable 2. **Average Number of Cigarettes Smoked per Day Among Daily and Someday Smokers Among Individuals Who Were Current Smokers by Cancer Type: National Health Interview Survey, United States, 2017
