# Supplementary material for: Smoking Behaviors in Survivors of Smoking-Related and Non–Smoking-Related Cancers
Source: JAMA Netw Open. 2020 Jul 2;3(7):e209072. doi: 10.1001/jamanetworkopen.2020.9072 (PMC7333020; doi:10.1001/jamanetworkopen.2020.9072)
Supplement: Supplement. — eTable 1. Age-Adjusted Prevalence of Daily and Someday Smokers Among Individuals Who Were Current Smokers by Cancer Type: National Health Interview Survey, United States, 2017 eTable 2. Average Number of Cigarettes Smoked per Day Among Daily and Someday Smokers Among Individuals Who Were Current Smokers by Cancer Type: National Health Interview Survey, United States, 2017 [file jamanetwopen-3-e209072-s001.pdf]

Supplementary Online Content

Gritz ER, Talluri R, Fokom Domgue J, Tami-Maury I, Shete S. Smoking behaviors in survivors of smoking-related and non–smoking-related cancers. *JAMA Netw Open*. 2020;3(7):e209072. doi:10.1001/jamanetworkopen.2020.9072

eTable 1. Age-Adjusted Prevalence of Daily and Someday Smokers Among Individuals Who Were Current Smokers by Cancer Type: National Health Interview Survey, United States, 2017

eTable 2: Average Number of Cigarettes Smoked per Day Among Daily and Someday Smokers Among Individuals Who Were Current Smokers by Cancer Type: National Health Interview Survey, United States, 2017

This supplementary material has been provided by the authors to give readers additional information about their work.

© 2020 Gritz ER et al. *JAMA Network Open.*

eTable 1: Age-Adjusted Prevalence of Daily and Someday Smokers Among Individuals Who Were Current Smokers by Cancer Type: National Health Interview Survey, United States, 2017

Cancer Type	Observed Numbers	Survey Weighted Numbers	Daily Smokers<br><br>Percentage (95% CI)	Someday Smokers<br><br>Percentage (95% CI)
**All current smokers**	4015	34284673	75.05 (73.23 - 76.78)	24.95 (23.22 - 26.77)
**Participants with cancer**	372	2573558	75.19 (63.54 - 84.06)	24.81 (15.94 - 36.46)
**Participants without cancer**	3637	31679815	74.74 (72.83 - 76.56)	25.26 (23.44 - 27.17)
**Smoking-related cancers**	145	978888	74.83 (61.67 - 84.6)	25.17 (15.40 - 38.33)
**Non-smoking– related cancers**	251	1738353	79.89 (69.89 - 87.18)	20.11 (12.82 - 30.11)
**Bladder***	15	83946	87.73 (-)	12.27 (-)
**Blood***	2	10553	49.80 (-)	50.20 (-)
**Bone**	1	3938	100.00 (-)	0.00 (-)
**Brain**	6	47412	83.24 (-)	16.76 (-)
**Breast**	51	358935	86.16 (74.12 - 93.12)	13.84 (6.88 - 25.88)
**Cervix***	56	407523	77.40 (68.4 - 84.41)	22.60 (15.59 - 31.6)
**Colon***	17	99414	43.91 (21.08 - 69.65)	56.09 (30.35 - 78.92)
**Esophagus***	1	3537	100.00 (-)	0.00 (-)
**Gallbladder**	0	0	-	-
**Kidney***	17	126170	96.23 (-)	3.77 (-)
**Larynx***	0	0	-	-
**Leukemia***	6	42537	64.98 (-)	35.02 (-)
**Liver***	5	20409	100.00 (-)	0.00 (-)
**Lung***	15	85594	88.60 (41.75 - 98.83)	11.40 (1.17 - 58.25)
**Lymphoma**	4	18207	100.00 (-)	0.00 (-)
**Melanoma**	17	131222	99.28 (57.87 - 99.99)	0.72 (0.01 - 42.13)
**Mouth***	3	28571	79.50 (-)	20.50 (-)
**Ovary**	13	89568	67.01 (14.99 - 95.9)	32.99 (4.10 - 85.01)
**Pancreas***	2	11552	100.00 (-)	0.00 (-)
**Prostate**	42	308265	86.99 (71.1 - 94.78)	13.01 (5.22 - 28.90)
**Rectum***	3	18573	66.93 (-)	33.07 (-)

© 2020 Gritz ER et al. *JAMA Network Open.*

**Skin1 (non melanoma)**	50	333628	80.25 (67.93 - 88.62)	19.75 (11.38 - 32.07)
**Skin2 (DK kind)**	29	173454	79.01 (34.58 - 96.4)	20.99 (3.60 - 65.42)
**Soft tissue**	3	17570	100.00 (-)	0.00 (-)
**Stomach***	6	49622	52.67 (-)	47.33 (-)
**Testis**	2	28620	68.79 (-)	31.21 (-)
**Throat***	2	17917	100.00 (-)	0.00 (-)
**Thyroid**	8	35964	87.18 (9.25 - 99.78)	12.82 (0.22 - 90.75)
**Uterus**	20	139346	69.53 (32.37 - 91.58)	30.47 (8.42 - 67.63)
**Other**	22	160640	93.67 (47.48 - 99.59)	6.334 (0.41 - 52.52)

*:Smoking-related cancers; DK: don't know; -: not estimable due to low sample size

© 2020 Gritz ER et al. *JAMA Network Open.*

eTable 2: Average Number of Cigarettes Smoked per Day Among Daily and Someday Smokers Among Individuals Who Were Current Smokers by Cancer Type: National Health Interview Survey, United States, 2017

**Cancer Type**	**Observed Numbers**	**Survey Weighted Number**	**Someday Smokers**	**Daily Smokers**
			**Mean (SD)**	**Mean (SD)**
**All current smokers**	4015	34284673	4.61(4.23-5.00)	13.66(13.28-14.04)
**Participants with cancer**	372	2573558	5.23(3.95-6.52)	13.56(12.69-14.43)
**Participants without cancer**	3637	31679815	4.58(4.18-4.98)	13.66(13.25-14.08)
**Smoking-related cancers**	145	978888	3.62(2.44-4.8)	13.82(12.39-15.26)
**Non-smoking– related cancers**	251	1738353	5.62(4-7.24)	13.32(12.28-14.36)
**Bladder***	15	83946	4.61(3.95-5.27)	11.01(5.80-16.22)
**Blood***	2	10553	2.00(-)	20.00(-)
**Bone**	1	3938	-	11.00(-)
**Brain**	6	47412	3.00(-)	14.18(9.72-18.63)
**Breast**	51	358935	4.45(3.05-5.86)	11.1(8.94-13.25)
**Cervix***	56	407523	3.72(2.7-4.73)	14.18(12.11-16.24)
**Colon***	17	99414	6.26(1.57-10.95)	15.05(9.60-20.51)
**Esophagus***	1	3537	-	10.00(-)
**Gallbladder**	0	0	-	-
**Kidney***	17	126170	3.00(-)	13.34(9.81-16.87)
**Larynx***	0	0	-	-
**Leukemia***	6	42537	4.00(-)	24.29(16.72-31.85)
**Liver***	5	20409	-	9.12(3.62-14.61)
**Lung***	15	85594	1.87(1.09-2.65)	10.51(7.49-13.54)
**Lymphoma**	4	18207	-	13.84(9.44-18.24)
**Melanoma**	17	131222	2.00(-)	14.93(10.25-19.6)
**Mouth***	3	28571	2.00(-)	13.97(9.21-18.74)
**Ovary**	13	89568	4.28(3.88-4.67)	11.29(8.52-14.05)
**Pancreas***	2	11552	-	24.04(3.33-44.74)
**Prostate**	42	308265	5.35(3.25-7.45)	12.53(9.48-15.58)
**Rectum***	3	18573	3.00(-)	7.12(2.41-11.82)
**Skin1 (non melanoma)**	50	333628	5.42(2.56-8.28)	12.12(10.07-14.16)
**Skin2 (DK kind)**	29	173454	6.05(3.66-8.43)	15.36(11.79-18.93)
**Soft tissue**	3	17570	-	9.24(7.74-10.73)
**Stomach***	6	49622	2.00(-)	10.00(-)
**Testis**	2	28620	4.00(-)	15.00(-)
**Throat***	2	17917	-	15.39(8.51-22.28)
**Thyroid**	8	35964	5.00(-)	12.58(6.07-19.09)
**Uterus**	20	139346	14.49(0.49-28.49)	14.18(10.47-17.88)
**Other**	22	160640	3.00(-)	15.69(13.19-18.19)

*:Smoking-related cancers; DK: don't know; -: not estimable due to low sample size

© 2020 Gritz ER et al. *JAMA Network Open.*
